# Nutrition and physical activity randomized control trial in child care centers improves knowledge, policies, and children’s body mass index

**DOI:** 10.1186/1471-2458-14-215

**Published:** 2014-03-01

**Authors:** Abbey Alkon, Angela A Crowley, Sara E Benjamin Neelon, Sherika Hill, Yi Pan, Viet Nguyen, Roberta Rose, Eric Savage, Nina Forestieri, Linda Shipman, Jonathan B Kotch

**Affiliations:** 1 Department of Family Health Care Nursing, University of California, San Francisco, School of Nursing, San Francisco, California, USA; 2 School of Nursing, Yale University, Orange, Connecticut, USA; 3 Department of Community and Family Medicine, Duke University Medical Center and Duke Global Health Institute, Durham, North Carolina, USA; 4 Division of Child and Family Mental Health and Developmental Neuroscience, Duke University, Durham, North Carolina, USA; 5 Frank Porter Graham Child Development Institute, The University of North Carolina at Chapel Hill, Chapel Hill, North Carolina, USA; 6 Department of Maternal and Child Health, The University of North Carolina at Chapel Hill, Chapel Hill, North Carolina, USA; 7 Department of Maternal and Child Health, CB# 7445 Rosenau Hall, The University of North Carolina at Chapel Hill, Chapel Hill, North Carolina 27599-7445, USA

**Keywords:** Child care, Nutrition, Physical activity, Body mass index, Child care health consultants, Obesity, Overweight

## Abstract

**Background:**

To address the public health crisis of overweight and obese preschool-age children, the Nutrition And Physical Activity Self Assessment for Child Care (NAP SACC) intervention was delivered by nurse child care health consultants with the objective of improving child care provider and parent nutrition and physical activity knowledge, center-level nutrition and physical activity policies and practices, and children’s body mass index (BMI).

**Methods:**

A seven-month randomized control trial was conducted in 17 licensed child care centers serving predominantly low income families in California, Connecticut, and North Carolina, including 137 child care providers and 552 families with racially and ethnically diverse children three to five years old. The NAP SACC intervention included educational workshops for child care providers and parents on nutrition and physical activity and consultation visits provided by trained nurse child care health consultants. Demographic characteristics and pre - and post-workshop knowledge surveys were completed by providers and parents. Blinded research assistants reviewed each center’s written health and safety policies, observed nutrition and physical activity practices, and measured randomly selected children’s nutritional intake, physical activity, and height and weight pre- and post-intervention.

**Results:**

Hierarchical linear models and multiple regression models assessed individual- and center-level changes in knowledge, policies, practices and age- and sex-specific standardized body mass index (zBMI), controlling for state, parent education, and poverty level. Results showed significant increases in providers’ and parents’ knowledge of nutrition and physical activity, center-level improvements in policies, and child-level changes in children’s zBMI based on 209 children in the intervention and control centers at both pre- and post-intervention time points.

**Conclusions:**

The NAP SACC intervention, as delivered by trained child health professionals such as child care health consultants, increases provider knowledge, improves center policies, and lowers BMI for children in child care centers. More health professionals specifically trained in a nutrition and physical activity intervention in child care are needed to help reverse the obesity epidemic.

**Trial registration:**

National Clinical Trials Number NCT01921842

## Background

Over the last decade, the steady increase in the prevalence of overweight and obesity in young children has created a public health crisis. The National Health and Nutrition Examination Survey in 2009–2010 found that 26.7% of two to five year olds were either overweight or obese [1]. Compared to children with healthy weights, children who are overweight or obese at three to five years of age are five times more likely to be overweight or obese in adolescence [2] and are at greater risk of chronic health problems later in life [3]. Interventions to address the obesity epidemic can reach the majority of young children if they are delivered through child care programs, where over 60% of preschool-age children in the United States (U.S.) spend an average of 30 hours per week [4-6].

Many studies conducted in child care centers show that children are not getting the recommended number of healthy foods and sufficient time for physical activity. Several nutrition and physical activity studies of child care programs showed that the majority did not have written policies on nutrition and physical activity, and those that had written policies did not meet current national recommendations [7-10]. A study of meals served to 117 two to five year olds in 20 child care centers found that children did not consume the recommended amounts of whole grains, fruits, or vegetables and exceeded recommended amounts of saturated fats and sugar [11].

Child care providers frequently rely on their own nutrition and physical activity habits rather than their knowledge of national recommendations, such as *Caring for our Children: National Health and Safety Performance Standards; Guidelines for Early Care and Education Program*s [12] or the Institute of Medicine (IOM) report [13] on health and safety policies for child care programs. At the same time, healthy child care center food policies, by themselves, do not necessarily translate into healthy eating practices [14]. Studies have shown that, with the addition of professional training, child care providers can effectively implement childhood obesity prevention practices [15,16].

Improving the diets of preschool-age children is a critical component of preventing unhealthy weight gain early in life. According to the 2002 and 2008 U.S. representative sample of 3,273 preschool-age children in the Feeding Infants and Toddlers Studies, young children consumed diets high in saturated fats or added sugars and low in dietary fiber [17]. In another U.S. nationally representative sample of 2,442 children two to eight years of age, children with diets high in energy density, such as foods with added sugars and fats compared to fruits and vegetables, were more likely to be overweight or obese rather than normal weight [18]. Furthermore, many preschool-age children’s beverage intake does not meet current dietary recommendations. In a U.S. representative sample of two to five year old children, a 24-hour dietary recall revealed that high sugar beverages were consumed by nearly half of the children - 46% drank 12 ounces of whole milk, 44% drank 5 ounces of fruit-flavored drinks, and 39% drank 3 ounces of soda on average in a given day [19].

Addressing physical activity in child care programs is also important in preventing early childhood obesity. Research studies have shown that preschool-age children in child care centers do not regularly engage in the recommended 60 minutes of moderate-to-vigorous physical activity each day [8,13], while other studies found they spend the majority of their time in sedentary behavior [20-22]. One study found that children spend more time in moderate-to-vigorous physical activity when child care programs have moveable playground equipment (e.g., balls, tricycles), lower use of electronic media, and larger playgrounds compared to programs without these characteristics [23].

Intervention studies in child care centers in the U.S. have had mixed results in decreasing the prevalence of children who are overweight or obese. Only two out of seven intervention studies achieved this goal [24,25]. The 14-week Hip-Hop to Health Jr. Obesity Prevention Effectiveness Trial for African American preschool-age children in child care centers reported significant decreases in body mass index (BMI) in children in the intervention versus control groups when the program was delivered by trained early childhood educators [25]. “Eat Healthy, Stay Active”, a six-month, quasi-experimental pilot intervention consisting of educational programs and activities for parents, staff and preschool-age children in six Head Start programs, showed a statistically significant decrease in child BMI and percent of obese children [24]. The Head Start Program is a U.S. federally-funded program that promotes the school readiness of children ages birth to five from low-income families by enhancing their cognitive, social and emotional development. Other intervention studies showed no change in children’s BMI [26-30].

In a study of the impact of the Child and Adult Care Food Program, there was no statistically significant difference in weight-for-height percentile of preschool children in one urban center participating in the program compared to children who brought their meals and snacks from home [29]. Also, in a culturally-tailored obesity intervention with classroom-based movement activities, staff development and peer-led parent education posters on nutrition and physical activity for Head Start programs serving primarily Mexican-American children, there was no significant difference in BMI between the children in the intervention or control centers [27]. The children in the two Head Start centers participating in a six-month intervention of staff and parent trainings and activities showed no significant decrease in standardized BMIs (zBMIs) compared to same-age children in one comparison Head Start center [28]. In Healthy & Ready to Learn, a randomized control trial in four Head Start programs with a 24 week multi-level intervention program comprised of parent and teacher education and child activities, there were no significant changes in BMI in the intervention versus control programs [30].

The Nutrition And Physical Activity Self-Assessment for Child Care (NAP SACC) is an intervention designed to enhance nutrition and physical activity environments in child care settings by improving the nutritional quality of food and beverages, the amount and quality of physical activity, staff-child interactions, and center nutrition and physical activity policies and practices [7,16,31]. The program has been used by a number of states [32] and incorporated into the U.S. public health campaign Let’s Move [33]. The program was initially pilot-tested by trained nurse child care health consultants (CCHCs) in North Carolina in a randomized, controlled study [16]. At the time of our study, NAP SACC had been associated with positive environmental nutrition and physical activity outcomes in a variety of child care programs, but there were no published studies that reported changes in children’s BMI.

The purpose of this study was to evaluate the impact of the NAP SACC intervention conducted by trained nurse child care health consultants in licensed child care centers in three states. CCHCs are child health professionals with specialized training in child care health and safety issues [34,35]. They conduct health and safety assessments, provide educational workshops in child care, consult with the directors on health and safety issues, and provide resources to help the center improve the quality of their health and safety policies and practices [36,37]. This paper will address the following objectives:

1. To determine if the NAP SACC workshops as delivered by nurse CCHCs improve child care providers’ or parents’ nutrition and physical activity knowledge.

2. To determine if child care center participation in the NAP SACC intervention delivered by nurse CCHCs improves the number and quality of written nutrition and physical activity policies, nutrition and physical activity practices, and children’s BMI.

## Methods

**Figure 1:**
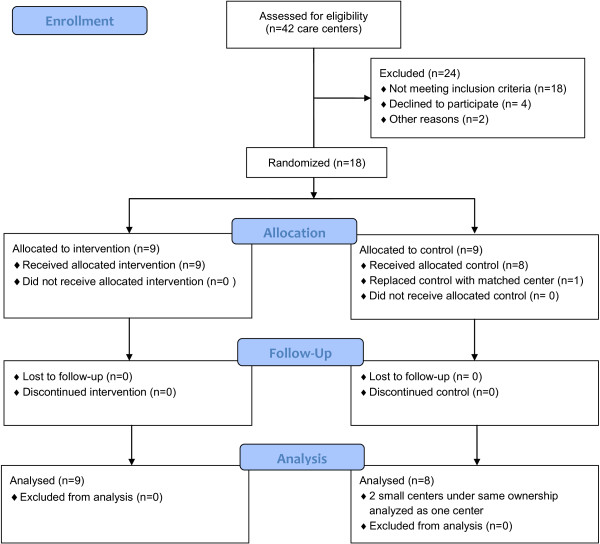
CONSORT flow diagram.

A seven-month (2009–2010) randomized control trial (RCT) was conducted in three states, California (CA), Connecticut (CT), and North Carolina (NC). Forty-two child care centers were recruited, of which 24 centers did not meet the inclusion criteria. Inclusion criteria included English-speaking director, on-site kitchen, racial/ethnic diversity among the children, participation by at least 60% of families, and a population of children in care primarily comprised of low-income children between the ages of three and five years of age. None of the centers enrolled in the study had participated in the NAP SACC program previously. One control center which withdrew when it was unable to complete the required number of study questionnaires was replaced with a matched center prior to intervention. Exclusion criteria for enrolling children included chronic illnesses or conditions that affected nutritional status, severe food allergies, gastrointestinal disorders or mobility impairment.Previously trained nurse CCHCs in each of the three states were hired for the purposes of this study. All received additional training in the NAP SACC intervention from one of the co-investigators. CCHCs from CA and NC recruited the convenience sample centers for their respective states while CT centers were recruited by the CT principal investigator, who is also a CHCC. The centers were matched on size and the proportion of children eligible for income subsidies and then randomly assigned to the NAP SACC intervention or control group. Six centers were enrolled in each state. In CT, two small control centers under the same ownership were merged for the analysis. A total of 17 centers had complete data, including nine intervention and eight control centers (Figure 1).

Each of the centers received $500 for its participation in the study. The intervention center directors were asked to purchase equipment or supplies to support physical activity. The control centers received the delayed NAP SACC intervention in year two of the study.

### NAP SACC intervention

The CCHCs facilitated five, one-hour NAP SACC workshops for child care providers and other staff (e.g., cooks, administrators) at each of the intervention centers on (1) childhood obesity, (2) healthy eating for young children, (3) physical activity for young children, (4) personal health and wellness, and (5) working with families to promote healthy behaviors. Seven of the intervention centers also received the parent workshop, “Raising Healthy Kids.” The workshops were held in the child care centers. In addition, the CCHCs worked with the center directors to write or update the center’s nutrition and physical activity policies. They also provided at least monthly on-site consultations and additional phone or email consultations and distributed posters and information sheets on nutrition and physical activities [16]. The posters were displayed in the child care centers, and the information sheets were given to the child care providers and parents. All of the materials presented and written for the project supported NAP SACC’s best practices [38]. Examples of some common issues addressed during the consultation visits were the type of milk served, healthy snacks, and ideas for structured physical activity.

### Measurement

Data collection occurred at baseline and seven months post-intervention at all centers. In each state a research assistant blinded to group assignment completed the center’s written policy assessments, center-level observational measures, and child-level height and weight measurements. One additional research assistant was trained by a co-investigator to complete the NAP SACC measures of nutritional intake and physical activity based on observations of individual children in all three states. This research assistant was blinded to group assignment and observed and recorded the foods offered at meals and snacks and the physical activity of a randomly selected group of children in each of the 17 enrolled centers. A 90% inter-rater reliability with the co-investigator was achieved before baseline data collection was initiated.

#### Demographics

Center directors, child care providers and parents completed demographic questionnaires, including information on ethnicity, education, and employment. The family’s total yearly gross income was divided by the number of persons in the household to determine whether the family fell within the 2010 U.S. Federal Poverty Guidelines [39].

#### CCHC activity

In addition to the aforementioned measures, the CCHCs completed a daily encounter form to report on their consultation activities, content covered, mode of communication, and travel time.

#### Provider and parent knowledge

Child care director, provider, other staff, and parent knowledge were measured before and immediately after each workshop using four multiple choice questions per workshop. Examples of two knowledge questions were, “How many minutes of active play each day do health professionals recommend for 3–5 year olds? (a) 30 minutes (b) 45 minutes (c) 60 minutes (d) 90 minutes (e) 120 minutes” and “Which of the following food groups should be eaten regularly? (a) whole grains (b) low fat dairy products (c) lean meat and beans (d) all of the above.” The questionnaires were developed by the study staff for this study, and content validity was assessed by the investigators. No psychometric tests were conducted. Separate mean scores were calculated for the pre- and post-workshop questionnaires for each intervention center and then analyzed by change over time.

#### Nutrition and physical activity policies

The policies on nutrition (11 items), sanitation for food preparation and food service (9 items), and physical activity (4 items) were evaluated by trained, blinded research assistants using the standardized California Childcare Health Program (CCHP) Health and Safety Policies Checklist [36] to determine if the center’s written policies adhered to *Caring for Our Children: National Health and Safety Performance Standards; Guidelines for Out-of-Home Child Care Program*s, Second Edition (NHS) [40]. Examples of some of the content required in a high quality policy on nutrition include: (1) healthy beverages are available all day, (2) menus are varied, (3) foods from different cultural groups are offered, (4) three week cycles of menus are planned for, and (5) child care providers eat with the children, provide family style meals, and encourage conversation during meals. To summarize the data collection procedures, each center’s written policy was first rated as present or not. If the written policy was present, individual components of the policy were each rated as yes (present) or no (not present) based on specific NHS content. Each policy was then summarized as a mean calculated as the number of components present divided by the number of components rated. The mean policy scores were calculated for each center and then aggregated by treatment or control group. This policy measurement technique was used in another study and shown to be a valid measure of the effect of CCHC interventions on child care center environments [41].

#### Nutrition and physical activity practices

Center-level nutrition and physical activity practices were assessed for the child care providers and children by different observational measures. A modified version of the Environmental Physical Activity Observation (EPAO) [7] was completed by each state’s research assistant to observe child care provider behaviors supporting healthy eating and physical activity in children. The measure included all of the eight items on the EPAO ‘eating occasions-staff behavior’ subscale and six out of seven items on the EPAO ‘physical activity-staff behavior’ subscale. The items were rated as either yes or no. The nutrition items included center-level observations of staff sitting with children during meals and snacks, talking with children about healthy foods, and eating the same food as the children. An example of a nutrition item is, “Did staff sit with children during lunch?” The six physical activity items included center-level observations of staff joining in active play, making positive comments about physical activity, providing prompts to increase physical activity, and offering formal physical activity lessons. An example of a physical activity item is “Did staff make positive statements about physical activity?” The observations were conducted over two to three hours during one day at each center. Although these items were modified from a reliable instrument, they were not previously validated in the format included in this study. Mean scores for the nutrition and physical activity scales were calculated for each center and then aggregated by treatment and control centers.

The Observation System for Recording Activity in Preschools (OSRAP) was completed by a trained and blinded research assistant to measure center-level physical activity over the course of a typical day. Eight children at each center, randomly selected by a statistician at the University of North Carolina at Chapel Hill (UNC), were observed in 15-second intervals for a total of 12 to 16 minutes per child; the observations were conducted over an eight hour day. Data were aggregated as the mean percent of physical activity intensity (1 = stationary to 5 = fast); types of activity (quiet, TV/screen, manipulative, gross motor); location of activity (classroom, gym, outdoor playground, outside general, eating, transition); context of activity (teacher directed, child initiated); interactions (none, child peer, provider, other adult); and prompts (none, increase activity, decrease activity). The OSRAP has been validated [41] and has been compared favorably with accelerometer data [9].

The Diet Observation in Child Care (DOCC) [11], which is a validated method for recording child-level nutrition in two to five year olds [23], was completed by one research assistant on the first three of the eight randomly selected children at each center to represent center-level nutrition. The trained and blinded observer recorded the types and portions of all foods and beverages served to target children during the observation day. The foods offered were categorized as grains, vegetables, fruit and 100% fruit juice, proteins, milk, snack foods, sweets, sweetened drinks, water, and condiments/seasonings. The percent of healthy foods offered within each category was calculated as the number of healthy foods served divided by the total number of foods offered in that category. An aggregate percent of healthy foods was calculated as the proportion of all whole grains, low fat meats and beans, dark green or orange vegetables, fruits and 100% fruit juice, and low- or non-fat milk served divided by all foods and beverages offered. The observations of food offered during lunch, not breakfast and snack, were included in the analysis because of greater consistency across the centers and minimal missing data since not all centers offered breakfast or snacks.

#### Body mass index (BMI)

The research assistants selected children at the pre-intervention period for height and weight measurements from center-specific randomly ordered lists of enrolled children. The total at the pre-intervention period, 268 of the 552 (49%) children enrolled in the study, was limited by availability of resources. Of the 268 children measured, 37% were from CA, 28% from CT and 35% from NC. However, to maximize the number of children with both pre- and post-intervention measurements, children who were measured pre-intervention were prioritized for post-intervention measurement, and as many as possible of the remaining available enrolled children who had not been measured at the pre-intervention period were measured post-intervention, bringing the total to 336, 34% from CA, 37% from CT, and 29% from NC. There were 211 children (63% of 336) with measurements at both the pre- and post-intervention periods, 38% from CA, 28% from CT, and 34% from NC. Two of these children were extreme outliers (greater than 3 standard deviations from the mean). Consistent with recommendations from the literature [42,43], these two were excluded from analyses, resulting in a total of 209 children.

The research assistants used a Seca™ 213 Portable Foldable Stadiometer to measure height. The Tanita HD 351 digital scale was calibrated daily and used to collect the children’s weights without their outer layers of clothing or shoes. BMI was calculated by dividing a child’s weight in kilograms by height in meters squared (kg/m^2^). The Centers for Disease Control and Prevention’s (CDC) program for SAS was used to calculate zBMI, an age- and sex-specific standardized measure of BMI [44]. The CDC defines categories of BMI percentile cut points as underweight (<5^th^ percentile), healthy (≥5^th^ to <85^th^ percentile), overweight (≥85^th^ to <95^th^ percentile) or obese (≥95^th^ percentile) [45].

### Statistical analysis

Descriptive statistics summarized the frequencies of the family, child, and center demographics and the CCHCs’ activities. T-tests or chi-square tests were conducted to compare the baseline demographic characteristics between the intervention and control centers and among the three states. Since there were significant differences in three demographic characteristics, state, parent education, and family poverty were included in subsequent hierarchical linear models (HLM) and multiple regression models as potential confounders. Pre-intervention *center-level* nutrition and physical activity policies, practices and observations (i.e., modified EPAO, OSRAP, DOCC, BMI) were compared between the intervention and control centers using independent samples T-tests. HLM models were conducted to assess *child-level* zBMI outcomes (accounting for clustering within center) and *center-level* provider and parent knowledge (accounting for repeated measures), controlling for state, parent education, and family poverty. Multiple regression models were used to assess *center-level* changes in nutrition and physical activity policies, nutrition and physical activity practices (i.e., modified EPAO, OSRAP, DOCC), and children’s zBMI from pre- to post-intervention, controlling for state, parent education and family poverty. The dummy variables for state (i.e., CA, CT) had NC as the reference category. Analyses were conducted with SAS 9.2 [46] and statistical significance was set a priori to < 0.05.

The Institutional Review Boards on Human Subject Research at UNC’s Gillings School of Global Public Health, Yale University’s School of Nursing, and the University of California, San Francisco’s School of Nursing approved the protocol and parent and child care provider consent forms. Center directors and parents provided written, informed consent to participate (or to have their children participate) in the study.

## Results

### Sample characteristics

The total sample included 552 three to five year old children and 137 child care providers (Table 1). The parents were 46% White, 17% Latino, 16% African American, 14% Asian, and 7% other ethnicity. The intervention and control centers demographic data were significantly different by parent ethnicity (chi-square(df) = 241.61(4), p < 0.0001), parent education (chi-square(df) = 26.85(1), p < 0.0001), household density (t statistic(df) = 2.72(537), p = 0.007), and family poverty (chi-square(df) = 24.24(2), p < 0.0001). At the center-level, parent education (t statistic(df) = 2.68(15), p = 0.02) and poverty level (t statistic(df) = -2.54(15), p = 0.02) were significantly different between the intervention and control centers. There were significant differences by state for child care provider education, ethnicity, and employment status, in addition to family poverty, parent ethnicity, education and household density. Therefore, all subsequent analyses controlled for state and for the center-level variables parent education and family poverty.

**Table 1:** Child, family, and center demographic characteristics by intervention and control

	**Intervention n = 260**	**Control n = 292**
	**N (%)**	**N (%)**
**Child characteristics**		
***Age in years***		
3	81 (31)	84 (29)
4	129 (50)	157 (54)
5	50 (19)	51 (17)
Total	260 (100)	292 (100)
***Sex***		
Male	140 (56)	146 (52)
Female	108 (44)	133 (48)
Total	248 (100)	279 (100)
**Family characteristics**		
***Parent ethnicity***	N (%)	N (%)
White*	141 (55)	109 (37)
African American	44 (17)	46 (16)
Hispanic, Latino	39 (15)	55 (19)
Asian*	22 (8)	55 (19)
Other*	12 (5)	26 (9)
Total	258 (100)	291 (100)
Parent education (primary caregiver)*		
Less than high school	59 (23)	128 (44)
High school and higher	198 (77)	163 (56)
Total	257 (100)	291 (100)
***Employment status (primary caregiver)***		
Working full time	189 (74)	211 (74)
Working part-time	37 (14)	49 (17)
Unemployed	5 (2)	14 (5)
Student*	14 (5)	6 (2)
Not working*	12 (5)	5 (2)
Total	257 (100)	285 (100)
***Government subsidies (chose all that apply)***		
Receive any subsidies	120 (46)	155 (53)
Food Stamps*	59 (23)	29 (10)
WIC	59 (23)	49 (17)
Medicaid	59 (23)	69 (24)
Child support	22 (8)	23 (8)
TANF	7 (3)	7 (2)
Housing	13 (5)	12 (4)
Other	19 (7)	24 (8)
Total	260	292
***Family poverty***		
Above 100% to 200% FPG	67 (30)	96 (35)
Above 200% FPG*	114 (50)	84 (30)
Total	226 (100)	278 (100)
Household density*	Mean (SD), N	Mean (SD), N
(# rooms/# people in household)	1.51 (0.74), 249	1.34 (0.72), 290
**Child care providers**	N = 76 providers	N = 61 providers
***Education***	N (%)	N (%)
Less than high school	3 (4)	4 (6)
High school graduate	11 (14)	8 (13)
Some college	41 (54)	34 (56)
Bachelor’s degree and higher	21 (28)	15 (25)
Total	76 (100)	61 (100)
***Ethnicity***		
White	33 (45)	29 (49)
African American	17 (23)	10 (17)
Hispanic, Latino	15 (21)	11 (19)
Asian	2 (3)	5 (8)
Other	6 (8)	4 (7)
Total	73 (100)	59 (100)
***Employment in years***	Mean (SD), N	Mean (SD), N
This center	6.19 (5.85), 65	6.29 (8.02), 57
Any center	11.26 (7.78), 63	10.70 (8.55), 53

*p < 0.05.

Note: T-tests were conducted for continuous variables and chi-square tests for categorical variables. There were missing data for some demographic variables.

FPG = Federal Poverty Guidelines; TANF = Temporary Assistance for Needy Families; WIC = Special Supplemental Nutrition Program for Women, Infants, and Children.

There were no significant differences in child care provider demographic characteristics between the intervention and control centers.

### CCHC activities

The nurse CCHCs conducted a mean (SD) of 11 (3) on-site visits and 8 (6) off-site consultations per center over the seven-month intervention, in addition to the provider and parent workshops. Each on-site consultation lasted a mean (SD) of 99 (55) minutes, and off-site consultation lasted a mean (SD) of 55 (64) minutes.

### Knowledge

Ninety child care directors, providers and staff, including cooks and administrators, and 45 parents participated in the workshops. The child care provider participation was 66% (90/137) with 83 participants for the Childhood Obesity workshops, 81 participants for the Healthy Eating workshops, 79 participants for the Physical Activity workshops, 78 participants for the Personal Health workshops and 77 participants for the Working with Families workshop. The parent participation rate was 20% (45/223). Seven out of the nine intervention centers provided parent workshops. There were statistically significant improvements in the center-level knowledge for child care providers and staff for four of the five provider workshops and for parents who attended the one parent workshop, controlling for state, parent education and family poverty level (Table 2).

**Table 2:** Center-level NAP SACC results for pre- and post-workshop knowledge questionnaires

**NAP SACC workshop topic**	**Pre-workshop mean (SD) range 0-4**	**Post-workshop mean (SD) range 0-4**	**t-statistic (df)**	**F-statistic (df)**	**p-value**
**Child care provider workshops (9 centers)**					
Childhood obesity	2.43 (0.14)	2.94 (0.39)	3.81 (4)	14.49 (1,4)	0.02
Healthy eating	2.05 (0.67)	3.63 (0.56)	7.05 (4)	49.67 (1,4)	0.00
Physical activity	2.93 (0.38)	3.05 (0.52)	0.83 (4)	0.68 (1,4)	0.46
Personal health	2.75 (0.49)	3.35 (0.49)	3.00 (4)	8.99 (1,4)	0.04
Working with families	3.52 (0.19)	3.83 (0.13)	3.41 (4)	11.64 (1,4)	0.03
**Parent workshop (7 centers)**					
Raising healthy kids	2.39 (0.8)	3.47 (0.46)	4.62 (2)	21.38 (1,2)	0.04

Note: HLM models controlling for state, parent education and family poverty status.

### Nutrition and physical activity policies

The intervention centers had significant increases in the quantity and quality of the nutrition and physical activity policies on the pre- versus post-intervention ratings compared to control centers (nutrition policies: R^2^ = .75, F statistic(df) = 6.63(5,11), p = 0.004; beta, 95% CI, t statistic = 5.36 (2.39,8.34), 3.97, p = 0.002; physical activity policies: R^2^ = .60, F statistic(df) = 3.33(5,11), p = 0.04; beta, 95% CI, t statistic = 3.69 (0.94,6.45), 2.95, p = 0.01). The intervention centers increased their mean nutrition policy scores from 0.89 to 5.17 (range 0 to 11), while there were no changes in the mean scores for the control centers. The intervention centers increased their mean physical activity policy scores from 0 to 2.82 (range 0 to 4), while there was no change in the mean scores for the control centers. There was no significant change in the policies for sanitation for food preparation and food service for either the intervention or control centers.

### Nutrition and physical activity practices

There were no significant changes in the child care provider eating and physical activities observed in the intervention versus control centers, controlling for state, parent education and poverty level, using the modified EPAO.

There were no significant changes from pre- to post-intervention in the type or intensity of physical activity between the intervention and control centers, controlling for state, parent education and poverty level, using the OSRAP. There were changes in the direction of decreased sedentary behaviors in the intervention centers. The children in the control centers increased their sedentary and/or quiet time from 53% to 58%, while the children in the intervention centers had a slight decrease from 60% to 56%, but these changes were not significant.

There were no significant changes from pre- to post-intervention in the children’s nutrition, controlling for state, parent education and poverty level, using the DOCC. There were some positive if non-significant changes in the foods offered, such as an 8% increase from the pre- to post-intervention periods in healthy foods in the intervention centers while the control centers had a 1% increase. The intervention centers also had a 10% increase of low- or non-fat milk offered compared to a 2% increase in the control centers. In addition, the intervention centers had a 17% increase in low fat meats and beans offered compared to an 8% decrease in the control centers.

### BMI

The child-level zBMI data showed there were no significant differences between the children in the intervention and control centers measured only at the pre-intervention period (n = 57) compared with children measured at both pre- and post-intervention periods (n = 209). A comparison of children with zBMI data only at the post-intervention period (n = 68) compared to children with zBMI data at both time points also showed no significant differences.

Among all 268 children measured at the pre-intervention period, there were no significant differences in the intervention versus control centers in the percent of children in the underweight, healthy, overweight or obese categories. Considering only those 209 children in the analytic sample, there were no significant changes from pre- to post-intervention in the percent of children in any of the four weight categories or in mean zBMIs within either the intervention or the control centers in bivariate analyses (Table 3).

**Table 3:** Frequency distribution of child-level BMI category and zBMI by intervention and control groups, pre- and post-intervention (n = 209)

	**Intervention**			**Control**		
**BMI Category**	**Pre-intervention N (%)**	**Post-intervention N (%)**	**p-value**	**Pre-intervention N (%)**	**Post-intervention N (%)**	**p-value**
Underweight	1 (1.01)	0 (0.00)	0.32	2 (1.82)	2 (1.82)	1.00
Healthy weight	58 (58.59)	62 (62.63)	0.56	68 (61.82)	72 (65.45)	0.58
Overweight	15 (15.15)	17 (17.17)	0.70	23 (20.91)	16 (14.55)	0.22
Obese	25 (25.25)	20 (20.20)	0.40	17 (15.45)	20 (18.18)	0.59
Total	99	99		110	110	
zBMI	M (SD), N	M (SD), N		M (SD), N	M (SD), N	
	0.75 (0.98), 99	0.71 (0.96), 99	0.79	0.61 (0.93), 110	0.67 (0.98), 110	0.66

Note: T-tests were conducted for BMI categories and zBMI means.

zBMI = age- and sex-specific standardized body mass index.

To test for a significant intervention versus control center difference in the changes in mean zBMI scores from pre- to post-intervention, *child level* analyses were conducted, including only those children with measurements at both points in time and excluding extreme outliers (n = 209). Using HLM with random center intercepts and controlling for state, parent education and family poverty, the difference in the mean *child-level* zBMI changes between intervention and control centers was significant: coeff(SE) -0.14(0.06); 95%CI (-0.26,-0.02); t-statistic(df)(-2.54); p = 0.02) (Table 4).

**Table 4:** Child-level zBMI change from pre- to post-intervention (n = 209)

**Variable**	**Coefficient estimate (SE)**	**95% CI**	**t-statistic (df)**	**p-value**
California (Reference: North Carolina)	0.11 (0.07)	(-0.04, 0.25)	1.59 (13)	0.14
Connecticut (Reference: North Carolina)	0.05 (0.07)	(-0.10, 0.19)	0.73 (13)	0.48
Parent education (reference: ≤ High School)	0.18 (0.06)	(0.06, 0.30)	2.93 (169)	0.004
Poverty level (reference: ≤ 100% FPG)	0.03 (0.06)	(-0.09, 0.15)	0.52 (169)	0.60
Intervention (reference: controls)	-0.14 (0.06)	(-0.26, -0.02)	-2.54 (13)	0.02
Intercept	-0.09 (0.08)	(-0.26, 0.07)	-1.22 (13)	0.24

Note: HLM (with random center intercept) controlling for state, parent education and family poverty status.

FPG = Federal Poverty Guidelines; zBMI = age- and sex-specific standardized body mass index.

To corroborate this finding and to be consistent with the analytic approach used for our other outcomes, multiple regression models controlling for state, parent education and poverty level were conducted at the *center-level*. The change in *center-level* mean zBMIs, the child-level data aggregated by center from pre- to post-intervention, also was significantly different in the intervention versus the control centers (Overall Model R^2^ = .42, F statistic(df) = 1.57(5); intervention coefficient (SE) = -.26(.1), p = 0.02) for the 209 children in the analytic sample. Model results in tabular format may be seen in Additional file 1. This decrease may be explained in part by the observation that, although there was an increase in healthy weight in both intervention and control centers, there was a net shift in the distribution of children from the obese to the overweight category in intervention centers, whereas the shift in the control centers appears to have gone in the opposite direction, from overweight to obese (Table 3).

## Discussion

This randomized control trial showed that a seven-month nutrition and physical activity intervention provided by nurse CCHCs significantly increased provider and parent knowledge, improved the number and quality of the nutrition and physical activity written policies, and decreased mean children’s zBMI in the intervention centers compared to matched control centers.

Other CCHC interventions have shown similar results. For example, a CCHC intervention in the state of Washington showing child care providers improved their knowledge of food safety after attending CCHC-led workshops [47]. There are few studies of interventions reporting on child care providers’ knowledge of nutrition and physical activity which support the IOM’s and other’s recommendations that child care providers be offered trainings to learn about ways to increase children’s physical activity and decrease their sedentary behavior [24,48]. Ongoing educational opportunities for child care providers should be designed to increase their self-efficacy, support their creativity, and increase their engagement in positive, healthy nutrition and physical activities.

### Nutrition and physical activity policies

Studies of CCHC interventions showed mixed results on changes in center’s written policies. One randomized control trial in California [37] and quasi-experimental studies in Washington [47] and North Carolina [36] showed statistically significant improvements in centers’ written policies, while two quasi-experimental studies showed no significant changes in health policies and emergency plan policies in Connecticut [49] and in a nationally representative group of Head Start centers [50].

It has been shown that child care policies on physical activity can have an impact on the level of moderate-to-vigorous physical activity and outdoor time provided for preschool-age children [51,52]. State child care licensing regulations and child care programs’ written policies are needed to ensure that children attending child care are given opportunities to engage in recommended structured and unstructured physical activities along with time outdoors to engage in moderate-to-vigorous physical activity. The IOM recommends that state child care regulations establish requirements for child care programs’ physical activity standards [13]. Similar policies are needed to support the provision of nutritious snacks and meals for preschool-age children attending child care centers.

### Nutrition and physical activity practices

Although our study showed decreases in zBMI, there was no corresponding significant increase in healthy lunches or moderate-to-vigorous physical activity. We did, however, observe a trend toward serving healthier lunches in the intervention versus control centers. In an earlier NAP SACC intervention conducted by 30 CCHCs in NC, there were significant and positive changes in healthy food as measured by the EPAO in the intervention centers compared to controls [7]. Other intervention studies, which did not include CCHCs, found mixed results. In a randomized control trial of high risk preschool-age children, a six-month family intervention of weekly parent and child groups for families with preschool-age children showed that children in the intervention group had lower zBMIs and consumed fewer calories from carbohydrates compared to the control group [53]. A quasi-experimental study of a center-based physical activity intervention for primarily Mexican-American children attending Head Start programs showed that children in the intervention groups consumed more fruits, vegetables, and low-fat milk [28]. On the other hand, a randomized control trial of a teacher-based weight control intervention for African American preschool children in child care centers found only one significant, positive change in dietary intake (percent of calories from saturated fat) for the intervention versus control centers [25].

The food served in our study’s child care centers was similar to that reported in observational studies from North Carolina [11], Oklahoma [54], and New York City [14]. These studies reported daily offerings of healthy foods such as fruits, non-fried vegetables, whole grains, and reduced-fat milk. In a survey of 1,583 Head Start centers nationally, 70% reported serving only nonfat or 1% fat milk, and 91% reported serving fruit and healthy vegetables every day [55]. Although other studies show similar foods being served in child care programs, there are inconsistent findings in intervention studies’ measurement of nutritional data and results.

The finding of high levels of sedentary behavior among our study children was consistent with other observational studies of preschool-age children’s physical activity in child care. One study showed that the children were sedentary 80% of the time [22], and another study showed that the children were sedentary 55% of the time [56]. These studies also showed that preschool-age children were engaged in moderate-to-vigorous physical activity only 3% [22] or 12% [56] of the time. Most of the studies of preschool-age children in child care centers show that the children were not engaged in the recommended 60 minutes of activity each day when in child care, including structured, unstructured, and moderate-to-vigorous physical activity [13,21]. Future evaluations of interventions in child care programs targeted at improving physical activity and increasing moderate-to-vigorous physical activity should use objective methods of physical activity measurement and evaluate both the processes and outcomes of the interventions [21].

### BMI

The magnitude of the decrease in mean children’s zBMI in the intervention centers compared to the control centers (0.14 in the child-level analysis, 0.26 in the center-level analysis) is consistent with observed child-level BMI or zBMI change in other nutrition and physical activity intervention studies with children of the same age. A systematic review of seven studies of obesity prevention programs lasting 12 or more weeks for children from infancy to five years of age that included appropriate BMI or zBMI data reported an overall child-level zBMI decrease of -0.26 (CI = -0.53 to 0.00) compared to controls [57]. One recent center-level quasi-experimental study with preschool-age children in child care showed a child-level zBMI decrease of 0.4 [24]. These center- or group-level interventions had similar or higher levels of zBMI change as our center-level intervention. In this study, we included the 209 children with usable, matched data at the pre- and post-intervention periods in the aggregated center-level data. This approach provided equal weights for each center in the model and was appropriate for our center-level design. Our nutrition and physical activity data were also collected at the aggregate level and summarized only at the center level. This different approach to the study design, data collection and analyses yielded similar effect sizes of center-level zBMI change as other studies reporting on child-level zBMI change.

### Limitations

Although this study found many positive effects of the NAP SACC intervention, there were some limitations. First, there were no repeated physical activity, nutrition, or height and weight observations conducted over multiple days at each pre- or post-intervention time point; thus the observations had limited test-retest reliability within each center. Second, there was a limited impact on parent nutrition and physical activity at home, since there was low parent attendance at the one parent workshop provided. Third, some centers had more child-level data and some states had more centers contributing to the final analyses. Fourth, some known confounders of children’s BMI, such as parent BMI and child-level accelerometer and sleep data were not collected. Fifth, although the pre-intervention heights and weights included children randomly selected in each site and data were missing at random, the unbalanced design may have underestimated our results. There were more children with post-intervention heights and weights, but only children with matched data were included in the center-level analyses of zBMI. Lastly, there was a historical cohort effect on both the intervention and control centers given obesity prevention campaigns at the national level (i.e., Let’s Move Child Care) and legislation (i.e., Healthy, Hunger-Free Kids Act) in the U.S. that promoted healthy nutrition and physical activity in child care during the study period.

## Conclusions

Our findings support the use of child health professionals such as CCHCs as effective change agents in child care centers through the use of the NAP SACC program. To increase child care centers’ access to CCHCs, federal, state and local funding and training are needed to strengthen the CCHC network. Federal government recommendations for child care programs support nutrition and physical activity programs that include the NHS recommendations and IOM policies [12,13] included in this project. These programs should be implemented and supported locally and statewide through regulations and quality rating and improvement systems [58]. Parent education is also important to ensure that there are consistent nutrition and physical activity practices at home and in the child care programs [59]. Several studies found that educational workshops are an effective approach to increase parent knowledge, but more research is needed to determine how to increase parent involvement.

Future studies should include a larger sample of centers and repeated measures of nutrition and physical activity practices, including objective accelerometer measures of physical activity and valid, standardized measure of both quantity and quality of the food consumed in child care programs over several days [60]. Future research also needs to focus on strategies to increase parent involvement in childhood obesity prevention. Interventions should be targeted to help child care providers and parents become role models for preschool-age children and help the children develop healthy nutrition behaviors [14]. To address the public health crisis of childhood obesity, we need to recruit, train and deploy more child health and nutrition professionals to provide nutrition and physical activity interventions in child care programs.

## Abbreviations

BMI: Body mass index, weight in kilograms divided by height in meters squared (kg/m^2^); CA: California; CCHC: Child care health consultant; CCHP: California childcare health program; CDC: Centers for disease control and prevention; CT: Connecticut; DOCC: Diet observation in child care; EPAO: Environmental physical activity observation; IOM: Institute of medicine; NAP SACC: Nutrition and physical activity self-assessment for child care; NHS: National health and safety standards; NC: North Carolina; OSRAP: Observation system for recording activity in preschools; RCT: Randomized control trial; UNC: University of North Carolina at Chapel Hill; zBMI: Age- and sex- specific standardized measure of BMI.

## Competing interests

The authors have no conflicts of interest or competing financial or non-financial interests to report.

## Authors’ contributions

AA contributed to all aspects of the project, led the CA arm of the project and wrote the manuscript. AC contributed to all aspects of the project, led the CT arm of the project and contributed to the manuscript. SEBN contributed to the design of the study, trained the CCHCs and research assistants, interpreted results, and contributed to the manuscript. SH interpreted the dietary intake findings and contributed to the interpretation of other results and to manuscript preparation. YP conducted the analyses and wrote the analysis reports. VN coordinated the project in North Carolina and wrote the progress reports for the North Carolina intervention. RR conducted the intervention in California and contributed to the analyses and manuscript preparation. ES contributed to data management and interpretation of the analyses. NF conducted supplemental data analyses and contributed to manuscript preparation. LS collected the observational data in the three states. JK developed the project design, obtained grant funding, led the NC arm of the study as well as supervised project implementation, data collection and data analysis, contributed to interpreting the data and to manuscript preparation and final editing for submission. All authors read and approved the final manuscript and agree to be accountable for all aspects of the work.

## Pre-publication history

The pre-publication history for this paper can be accessed here:

http://www.biomedcentral.com/1471-2458/14/215/prepub

## Supplementary Material

Additional file 1 Changes in children’s center-level zBMIs from pre- to post-intervention (n=17 centers). Click here for file

## References

[B1] Ogden C Carroll M Kit B Flegal K Prevalence of obesity and trends in Body Mass Index among US children and adolescents, 1999–2010 JAMA 2012 307 483 490 10.1001/jama.2012.40 22253364 PMC6362452

[B2] Nadar P O'Brien M Houts R Bradley J Belsky J Crosnoe R Friedman S Mei Z Susman E Identifying risk for obesity in early childhood Pediatrics 2006 118 e594 e601 10.1542/peds.2005-2801 16950951

[B3] Barlow S Expert Committee Recommendations regarding the prevention, assessment, and treatment of child and adolescent overweight and obesity: summary report Pediatrics 2007 120 Supplement 4 S164 S192 18055651 10.1542/peds.2007-2329C

[B4] Natale R Scott SH Messiah SE Schrack MM Uhlhorn SB Delamater A Design and methods for evaluating an early childhood obesity prevention program in the childcare center setting BMC Public Health 2013 13 78 10.1186/1471-2458-13-78 23356862 PMC3573935

[B5] Larson N Ward D Benjamin Neelon S Story M What role can child-care settings play in obesity prevention? A review of the evidence and call for research efforts J Am Diet Assoc 2011 111 1343 1362 10.1016/j.jada.2011.06.007 21872698

[B6] National Association of Child Care Resource and Referral Agencies (NACCRRA) Child Care in America: 2010 State Fact Sheets 2010 Washington DC

[B7] Ward D Benjamin SE Ammerman AS Ball SC Neelon BH Bangdiwala SI Nutrition and physical activity in child care: results from an environmental intervention Am J Prev Med 2008 35 4 352 356 10.1016/j.amepre.2008.06.030 18701236

[B8] Wolfenden L Neve M Farrell L Lecathelinais C Bell C Milat A Wiggers J Sutherland R Physical activity policies and practices of childcare centers in Australia J Paediatr Child Health 2009 47 73 76 20500433 10.1111/j.1440-1754.2010.01738.x

[B9] Trost S Messner L Fitzgerald K Roths B Nutrition and physical activity policies and practices in family child care homes Am J Prev Med 2009 37 537 540 10.1016/j.amepre.2009.09.020 19944921

[B10] Benjamin Neelon S Vaughn A Ball S McWilliams C Ward D Nutrition practices and mealtime environments of North Carolina child care centers Childhood Obesity 2012 8 216 223 22799547 10.1089/chi.2011.0065

[B11] Ball SC Benjamin SE Ward DS Development and reliability of an observation method to assess food intake of young children in child care J Am Diet Assoc 2007 107 656 661 10.1016/j.jada.2007.01.003 17383271

[B12] American Academy of Pediatrics, American Public Health Association, National Resource Center for Health and Safety in Child Care and Early Education Caring for our Children: National Health and Safety Performance Standards; Guidelines for Early Care and Education Programs 2011 3 Elk Grove Village, IL

[B13] Institute of Medicine Glickman D, Parker L, Sim L, Del Valle Cook H, Miller E Accelerating Progress on Obesity Prevention: Solving the Weight of the Nation 2012 Washington, DC: The National Academies Press 24830053

[B14] Erinosho T Dixon L Young C Brotman L Hayman L Nutrition practices and children's dietary intakes at 40 child-care centers in New York City J Am Diet Assoc 2011 111 1391 1397 10.1016/j.jada.2011.06.001 21872704

[B15] Sigman-Grant M Christiansen E Fernandez G Fletcher J Johnson SL Branen L Price BA Child care provider training and a supportive feeding environment in child care settings in 4 states, 2003 Prev Chronic Dis 2011 8 A113 21843416 PMC3181186

[B16] Benjamin S Ammerman AS Sommers JK Dodds JM Neelon BH Ward D Nutrition and physical activity self-assessment for child care (NAP SACC): results from a pilot intervention J Nutr Educ Behav 2007 39 3 142 149 10.1016/j.jneb.2006.08.027 17493564

[B17] Butte N Fox MK Briefel RR Siega-Riz AM Dwyer JT Deming DM Reidy KC Nutrient intakes of US infants, toddlers, and preschoolers meet or exceed dietary reference intakes J Am Diet Assoc 2010 110 12 Suppl S27 S37 21092766 10.1016/j.jada.2010.09.004

[B18] Vernarelli J Mitchell D Hartman R Rolls B Dietary energy density is associated with body weight status and vegetable intake in U.S. children J Nutr 2011 141 2204 2210 10.3945/jn.111.146092 22049295 PMC3223877

[B19] O'Connor T Yang S Nicklas T Beverage intake among preschool children and its effect on weight status Pediatrics 2006 118 e1010 e1018 10.1542/peds.2005-2348 17015497

[B20] Oliver M Schofield G Kolt G Physical activity in preschoolers: understanding prevalence and measurement issues Sports Med 2007 37 1045 1070 10.2165/00007256-200737120-00004 18027993

[B21] Reilly J Low levels of objectively measured physical activity in preschoolers in child care Med Sci Sports Exerc 2010 42 502 507 20068499 10.1249/MSS.0b013e3181cea100

[B22] Pate RR McIver K Dowda M Brown W Addy C Directly observed physical activity levels in preschool children J Sch Health 2008 78 438 444 10.1111/j.1746-1561.2008.00327.x 18651931

[B23] Dowda M Brown WH McIver KL Pfeiffer KA O'Neill JR Addy CL Pate RR Policies and characteristics of the preschool environment and physical activity of young children Pediatrics 2009 123 2 261 266 10.1542/peds.2008-2498 PMC2632768 19171578

[B24] Herman A Nelson B Teutsch C Chung P "Eat Healthy, Stay Active!": a coordinated intervention to improve nutrition and physical activity among Head Start parents, staff, and children Am J Health Promot 2012 27 1 e27 e36 10.4278/ajhp.110412-QUAN-157 22950932

[B25] Fitzgibbon M Stolley MR Van Horn L Kaufer-Christoffel K Dyer A Two-year follow-up results for Hip-Hop to Health Jr: a randomized controlled trial for overweight prevention in preschool minority children J Pediatr 2005 146 618 625 10.1016/j.jpeds.2004.12.019 15870664

[B26] Fitzgibbon M Stolley MR Schiffer LA Braunschweig CL Gomez SL Van Horn L Dyer A Hip-Hop to Health Jr. obesity prevention effectiveness trial: postintervention results Obesity 2011 19 994 999 10.1038/oby.2010.314 21193852 PMC3775499

[B27] Fitzgibbon M Stolley MR Schiffer L Van Horn L Kaufer-Christoffel K Dyer A Hip-Hop to Health Jr. for Latino preschool children Obesity 2006 14 9 1616 1625 10.1038/oby.2006.186 17030973

[B28] Yin Z Parra-Medina D Cordova A He M Trummer V Sosa E Gallion KJ Sintes-Yallen A Huang Y Wu X Acosta D Kibbe D Ramirez A Miranos! Look at us, we are healthy! An environmental approach to early childhood obesity prevention Childhood Obesity 2012 8 429 439 23061498 10.1089/chi.2012.0125 PMC3621338

[B29] Bruening K Gilbride J Passannante M McClowry S Dietary intake and health outcomes among young children attending 2 urban day-care centers J Am Diet Assoc 1999 99 1529 1535 10.1016/S0002-8223(99)00375-2 10608946

[B30] Winter S Sass D Healthy & Ready to Learn: examing the efficacy of an early approach to obesity prevention and school readiness J Res Child Educ 2011 25 304 325

[B31] Ammerman A Ward DS Benjamin SE Ball SC Sommers JK Molloy M Dodds JM An intervention to promote healthy weight: nutrition and physical activity self-assessment for child care (NAP SACC) theory and design Prev Chronic Dis 2007 4 1 12 PMC1955393 17572971

[B32] Drummond R Staten LK Sanford MR Davidson CL Ciocazan MM Khor K-N Kaplan F Steps to a healthlier Arizona. A pebble in the pond: the ripple effect of an obesity prevention intervention targeting the child care environment Health Promot Pract 2009 10 156S 167S 10.1177/1524839908331267 19454762

[B33] Let’s Move: America’s move to raise a healthier generation of kids[http://www.letsmove.gov/]

[B34] Cianciolo S Trueblood-Noll R Allingham P Health consultation in early childhood settings Young Children 2004 59 56 61

[B35] Crowley AA Child care health consultation: an ecological model J Soc Pediatr Nurs 2001 6 170 181 10.1111/j.1744-6155.2001.tb00241.x 11777330

[B36] Isbell P Kotch JB Savage E Gunn E Lu LS Weber DJ Improvement of child care programs’ health and safety policies, and practices, and children’s access to health care linked to child care health consultation NHSA Dialog: Res Pract J 2013 16 34 52

[B37] Alkon A Bernzweig J To K Wolff M Mackie J Child care health consultation improves health and safety policies and practices Acad Pediat 2009 9 366 370 10.1016/j.acap.2009.05.005 19640823

[B38] NAPSACC Nutrition and Physical Activity Self Assessment for Child Care[http://gonapsacc.org/]

[B39] Office of the Assistant Secretary for Planning and Evaluation Prior HHS poverty guidelines and Federal Register references[http://aspe.hhs.gov/poverty/figures-fed-reg.cfm]

[B40] American Academy of Pediatrics, American Public Health Association, National Resource Center for Health and Safety in Child Care Caring for our Children: National Health and Safety Performance Standards; Guidelines for Out-of-Home Child Care Programs 2002 2 Elk Grove Village, IL

[B41] Brown W Pfeiffer KA McIver KL Dowda M Almeida J Pate RR Assessing preschool children's physical activity: the observational system for recording physical activity in children - preschool version Res Q Exerc Sport 2006 77 167 176 16898273 10.1080/02701367.2006.10599351

[B42] Salomon K Respiratory sinus arrhythmia during stress predicts resting respiratory sinus arrhythmia 3 years later in a pediatric sample Health Psychol 2005 24 68 76 15631564 10.1037/0278-6133.24.1.68

[B43] Alkon A Boyce WT Davis NV Eskenazi B Developmental changes in autonomic nervous system resting and reactivity measures in Latino children from 6 to 60 months of age J Dev Behav Pediatr 2011 32 668 677 10.1097/DBP.0b013e3182331fa6 22008788

[B44] A SAS Program for the CDC Growth Charts[http://www.cdc.gov/nccdphp/dnpao/growthcharts/resources/sas.htm]

[B45] Kuczmarski F Ogden CL Guo SS Grummer-Strawn LM Flegal KM Mei Z Mei R Curtin FL Roche AF Johnson CL 2000 CDC Growth Charts for the Unisted States: methods and development Vital and Health Statistics Series 11 2002 246 1 190 12043359

[B46] SAS Institute, Inc SAS/STAT 9.2 User’s Guide 2009 2 Cary, NC

[B47] Organizational Research Services Pilot Evaluation Report, Executive Summary, Healthy Child Care Washington 2003 Seattle, WA

[B48] Institute of Medicine Early Childhood Obesity Prevention Policies 2011 Washington, DC: The National Academies Press 10.3945/an.111.001347 PMC3262615 22332102

[B49] Crowley A Kulikowich J Impact of training in child care health consultant knowledge and practice Pediatr Nurs 2009 35 93 100 19472672

[B50] Hanna H Mathews F Southward LH Cross GW Kotch J Blanchard T Cosby AG Use of paid child care health care consultants in early care and education settings: results of a national study comparing provision of health screening services among Head Start and non-Head Start centers J Pediatr Health Care 2012 26 427 435 10.1016/j.pedhc.2011.05.008 23099309

[B51] Copeland K Sherman S Kendeigh C Kalkwarf H Saelens B Societal values and policies may curtail preschool children's physical activity in child care centers Pediatrics 2012 129 265 274 10.1542/peds.2011-2102 22218842 PMC3269117

[B52] Copeland K Sherman SN Khoury JC Foster KE Saelens BE Kalkwarf HJ Wide variability in physical activity environments and weather related outdoor play policies in child care centers within a single county of Ohio Arch Pediat Adol Med 2011 165 435 442 10.1001/archpediatrics.2010.267 PMC3086945 21199969

[B53] Brotman L Dawson-McClure S Huang K-Y Theise R Kamboukos D Wang J Petkova E Ogedegbe G Early childhood family intervention and long-term obesity prevention among high-risk minority youth Pediatrics 2012 129 e621 e628 10.1542/peds.2011-1568 22311988 PMC3289522

[B54] Sisson S Campbell JE May KB Brittain DR Monroe LA Guss SH Ladner JL Assessment of food, nutrition, and physical activity practices in Oklahoma child-care centers J Acad Nutr Diet 2012 112 1230 1240 10.1016/j.jand.2012.05.009 22818731

[B55] Whitaker R Gooze R Hughes C Finkelstein D A national survey of obesity prevention practices in Head Start Arch Pediat Adol Med 2009 163 1144 1150 10.1001/archpediatrics.2009.209 19996052

[B56] Bower J Hales DP Tate DF Rubin DA Benjamin SE Ward DS The childcare environment and children's physical activity Am J Prev Med 2008 34 23 29 10.1016/j.amepre.2007.09.022 18083447

[B57] Waters E de Silva-Sanigorski A Burford BJ Brown T Campbell KJ Gao Y Armstrong R Prosser L Summerbell CD Interventions for preventing obesity in children Cochrane Database Syst Rev 2011 Issue 12. Art. No.: CD001871. DOI:10.1002/14651858.CD001871.pub3 10.1002/14651858.CD001871.pub3 22161367

[B58] Benjamin S Cradock A Walker E Slining M Gillman M Obesity prevention in child care: a review of U.S. state regulations BMC Public Health 2008 8 188 10.1186/1471-2458-8-188 18513424 PMC2438347

[B59] Mendez J How can parents get involved in preschool? Barriers and engagement in education by ethnic minority parents of children attending Head Start Cult Divers Ethn Min 2010 16 26 36 10.1037/a0016258 20099962

[B60] Ward D Vaughn A Story M Expert and stakeholder consensus on priorities for obesity prevention research in early care and education settings Childhood Obesity 2013 9 116 124 23506454 10.1089/chi.2013.9204 PMC3713439

