# Supplementary material for: Nutrition and physical activity randomized control trial in child care centers improves knowledge, policies, and children’s body mass index
Source: BMC Public Health. 2014 Mar 1;14:215. doi: 10.1186/1471-2458-14-215 (PMC3945995; doi:10.1186/1471-2458-14-215)
Supplement: Additional file 1 — Changes in children’s center-level zBMIs from pre- to post-intervention (n=17 centers). [file 1471-2458-14-215-S1.pdf]

**Additional File 1. Changes in children's center-level zBMIs from pre- to post-intervention (n=17 centers)**

| Variable | Coefficient estimate (SE) | 95% CI | t-statistic(df) | p-value |
|---|---|---|---|---|
| California (Reference: North Carolina) | 0.13 (0.10) | (-0.10,0.36) | 1.21(11) | 0.25 |
| Connecticut (Reference: North Carolina) | 0.15 (0.10) | (-0.08,0.37) | 1.47(11) | 0.17 |
| Parent education (reference: ≤High School) | 0.59 (0.31) | (-0.11,1.28) | 1.87(11) | 0.09 |
| Poverty level (reference: ≤100% FPG) | 0.03 (0.23) | (-0.47,0.54) | 0.15(11) | 0.88 |
| Intervention (reference: controls) | -0.26 (0.10) | (-0.47,-0.04) | -2.63(11) | 0.02 |
| Intercept | -0.36 (0.27) | (-0.96,0.24) | -1.32(11) | 0.22 |

Note: Multiple regression model controlling for state, parent education and family poverty status: Overall model $R^2$=.42, F statistic(df)=1.57(5), p=0.25
FPG = Federal Poverty Guidelines; zBMI = age- and sex-specific standardized body mass index
